# Biphasic calcium phosphates (BCP) of hydroxyapatite (HA) and tricalcium phosphate (TCP) as bone substitutes: Importance of physicochemical characterizations in biomaterials studies

**DOI:** 10.1016/j.dib.2016.11.080

**Published:** 2016-11-28

**Authors:** Mehdi Ebrahimi, Michael Botelho

**Affiliations:** Oral rehabilitation, Prince Philip Dental Hospital, Faculty of Dentistry, The University of Hong Kong, Hong Kong

**Keywords:** Biphasic calcium phosphate, Characterization, Study protocol, Bone tissue engineering

## Abstract

The data presented in this article are related to the research article entitled “Biphasic calcium phosphates bioceramics (HA/TCP): Concept, physicochemical properties and the impact of standardization of study protocols in biomaterials research” [1]. This article provides in depth study of BCP bone substitutes as valuable option in the field of tissue engineering. However, there are discrepancies in the literature regarding the ideal physicochemical properties of BCP and the ideal balance between different phase compositions for enhanced bone tissue engineering (M. Ebrahimi, M.G. Botelho, S.V. Dorozhkin, 2016; M. Ebrahimi, P. Pripatnanont, S. Suttapreyasri, N. Monmaturapoj, 2014) [1,2]. This is found to be mainly because of improper characterization of BCP bioceramics in basic studies and lack of standard study protocols in *in vitro* and *in vivo* research. This data article along with original article provide the basic data required for ideal characterization of BCP and other bioceramics in an attempt to provide basic standardized protocols for future studies.

**Specifications Table**

**Table:** 

Subject area	Bone tissue engineering
More specific subject area	Biphasic calcium phosphates bone substitutes
Type of data	Figures, graph, X-ray images and table
How data was acquired	Electronic data base (PubMed), systematic literature review
Data format	Analyzed
Experimental factors	N/A
Experimental features	Description of BCP; synthesis/characterizations.
Data source location	Prince Philip Dental Hospital, Faculty of Dentistry, The university of Hong Kong, Hong Kong
Data accessibility	Data are available with this article

**Value of the data:**

• To provide basic standard data for proper characterization of BCP and other bone substitutes.
• To encourage researchers to standardize their study protocols.
• To help in reducing the discrepancies among the findings of future studies.

## 1 Data

This paper presents the required data and examples on proper characterization of BCP. This can be applied to other similar materials in the field of bone tissue engineering [2]. Data on use of XRD (X-ray diffraction), SEM (scanning electron microscope), mechanical testing (MT) and other investigations have been provided.

**Fig. 1:**
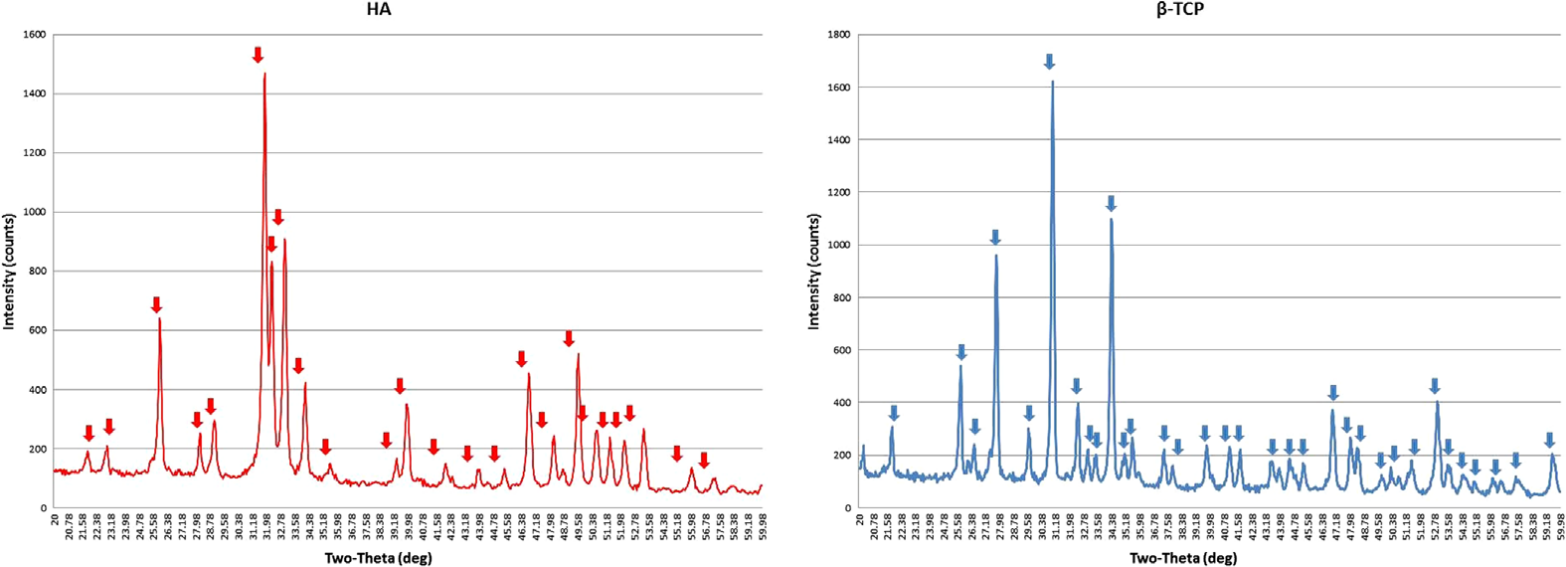
XRD pattern of pure HA and β-TCP. The main corresponding peaks are marked.

Fig. 1. XRD showing the crystallographic pattern and corresponding peaks of HA and β-TCP according to ICDD (International Center for Diffraction Data) database.

**Fig. 2:**
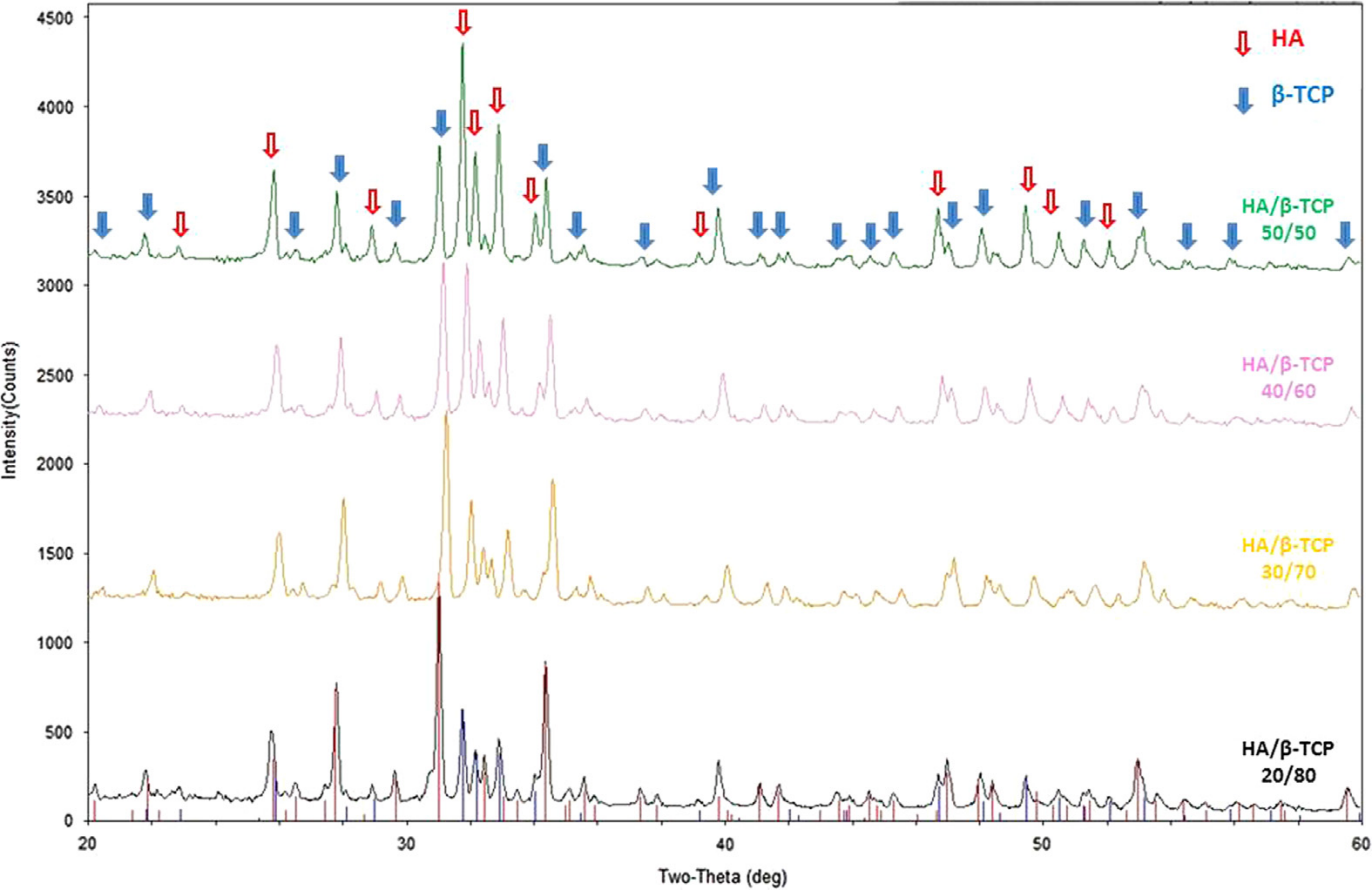
XRD pattern of BCP (HAp/β-TCP) at 50/50, 40/60 and 30/70 percentage composition ratio. The corresponding peaks of each phase are highlighted.

Fig. 2. XRD pattern of different composition ratios of BCP. The intensity and pattern of corresponding peaks change according to the relative composition ratio of HA/ β-TCP.

**Fig. 3:**
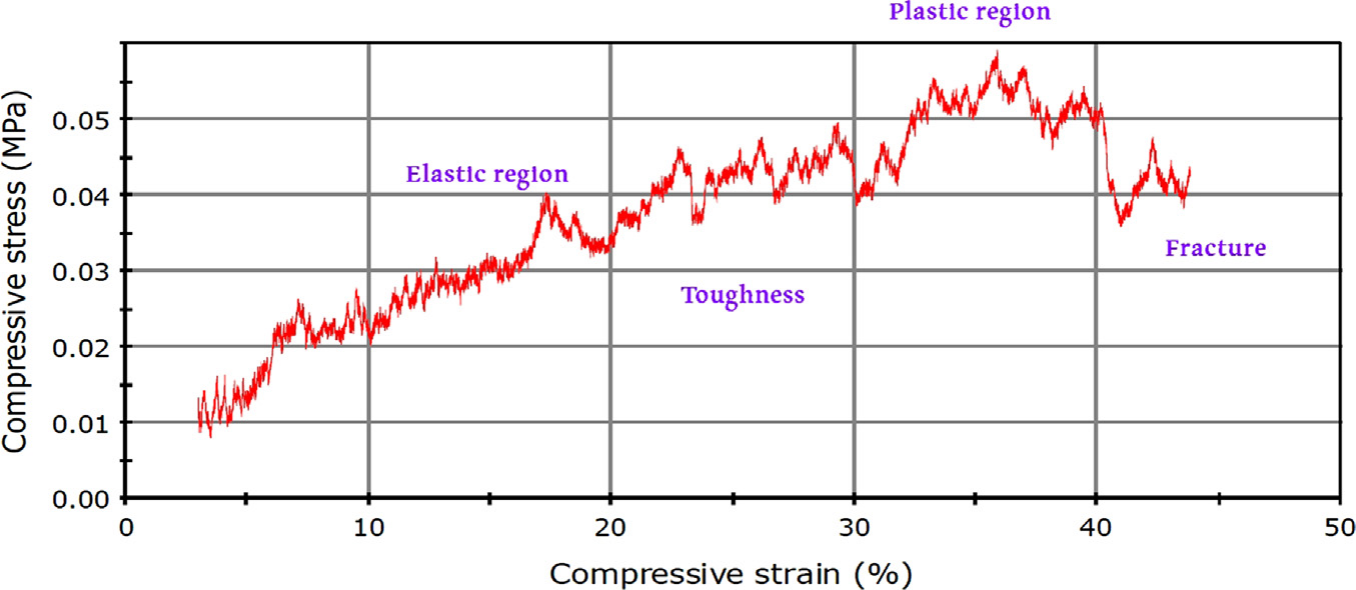
Typical stress–strain curve.

Fig. 3. SEM image of HA particles illustrating analysis of morphology and dimension.

**Fig. 4:**
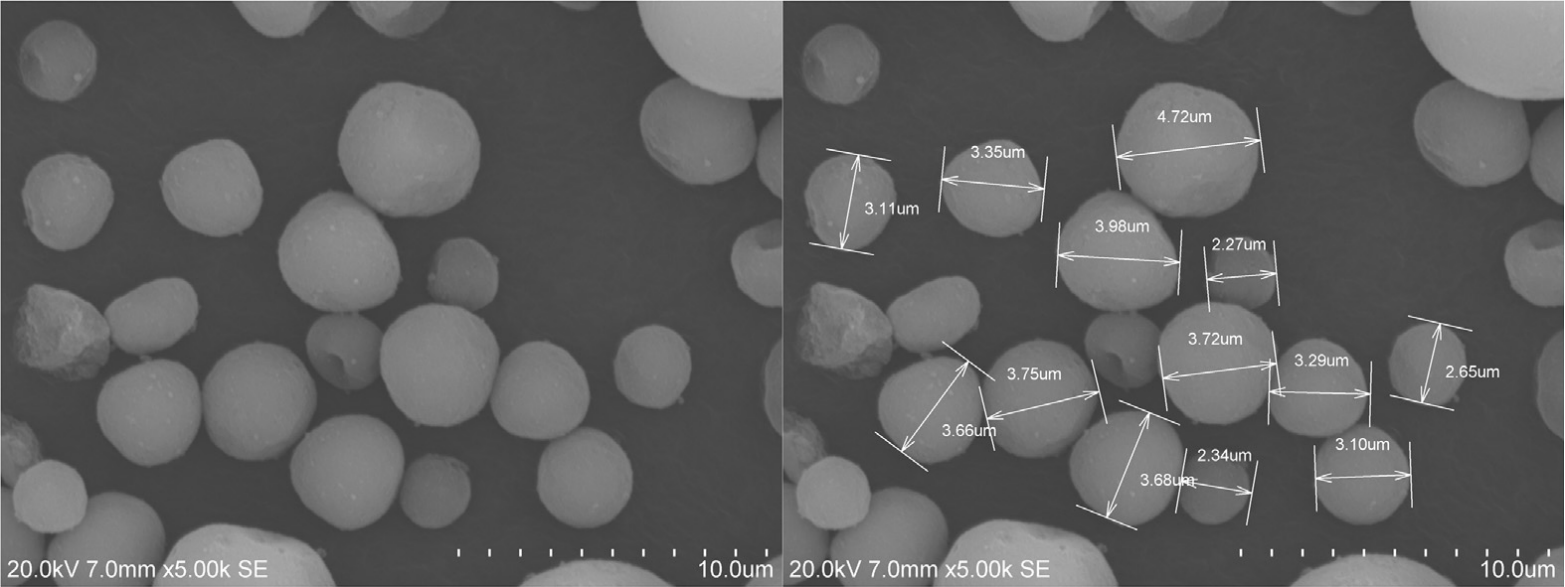
SEM images showing spherical HA particles and their dimensions.

Fig. 4. The stress–strain curves for the BCP scaffolds. The scaffold has an initial elastic region where the deformations are reversible (elastic deformation), followed by a plastic region before failure presented by a sudden drop in the cure which indicate irreversible change (fracture).

Table 1. Recommended investigations for characterization of BCP bioceramics and other bone substitute biomaterials.

## 2 Experimental design, materials and methods

An electronic data base search on PubMed was performed to recruit related literature on BCP including data on basic biomaterials science, synthesis and characterization. Interested readers are referred to full text of this review paper for comprehensive review and recommendations [1].

## Figures and Tables

**Table 1:** General recommendations for characterization techniques of BCP bioceramics and other biomaterials for bone tissue engineering.

**Test**	**Parameters**	**Standard unit**
XRD	To study the crystallography and corresponding peak phases and intensity comparing to the standard JPDC card for HA (090432) and TCP (090169).	Intensity/ 2θ degree
	Note 1: sintering >1250 °C may cause phase transformation within BCP [3].	
	Note 2: major peaks of BCP are located between 20–60° two theta degrees.	
	Note 3: recommended radiation is ≈ 40 kV and 30 mA in 0.02° steps from 20° to 60° (2θ) [2].	
PSA	To study accurate particle size distribution pattern using laser diffraction method.	µm, nm
		
SEM	To illustrate macro and microstructure images at different magnifications.	µm, nm
		magnifications, i.e.; x100, x500, x1000
	To evaluate roughness, pore size, geometry and total porosity (software program may be required).	
	To evaluate particle/grain size and geometry.	
	Note 1: recommended kV is ≈ 5–20.	
		
UTM	To measure mechanical properties, compressive strength, toughness and fracture resistance using stress–strain curve.	kPa, MPa, N
	Note 1: prepare enough scaffolds (*n*=3–5) to allow for mean calculation.	
	Note 2: prepare scaffolds with length twice width.	
	Note 3: use static or dynamic load cells (N) at a defined crosshead speed (mm/min). ASTM provides useful guidelines for different materials.	
	Note 4: pre-hydrated scaffold may give different readings than dry one [4]	
		
FTIR	For precise chemical composition and structural investigation of composite scaffolds.	wavenumber cm^−1^
	Note 1: recommended reading from 4000–400 wavenumber cm^−1^.	
		
TEM	To study spatial relationship between collagen and mineral phase.	nm
	To study crystalline diffraction pattern.	
	To study morphological feature of nanostructure.	
EDS	For chemical characterization and molar ratio (Ca/P) analysis.	Kα, KeV
XRF	For detailed analyses of molar ratio (Ca/P) and other elements.	Kα, KeV, nm
Micro-CT	For 2D and 3D image reconstruction and analysis of porosity and internal structure.	µm/ voxel

*Abbreviations:* XRD: X-ray diffractometer; PSA: Particle size analyzer; SEM: Scanning electron microscopy; UTM: Universal testing machine; FTIR: Fourier transform infrared spectroscopy; TEM: Transmission electron microscopy; EDS: Energy-dispersive X-ray spectroscopy; XRF: X-ray fluorescence analysis; Micro-CT: Micro computed tomography.
